# Supplementary material for: Atomic resolution cryo-EM structure of a native-like CENP-A nucleosome aided by an antibody fragment
Source: Nat Commun. 2019 May 24;10:2301. doi: 10.1038/s41467-019-10247-4 (PMC6534667; doi:10.1038/s41467-019-10247-4)
Supplement: Supplementary file 1 — Supplementary Information [file 41467_2019_10247_MOESM1_ESM.pdf]

Supplementary Information

Atomic resolution cryo-EM structure of a native-like CENP-A nucleosomes aided by an antibody fragment

Zhou et al.

Supplementary Figures: 1 – 14

Supplementary Table 1

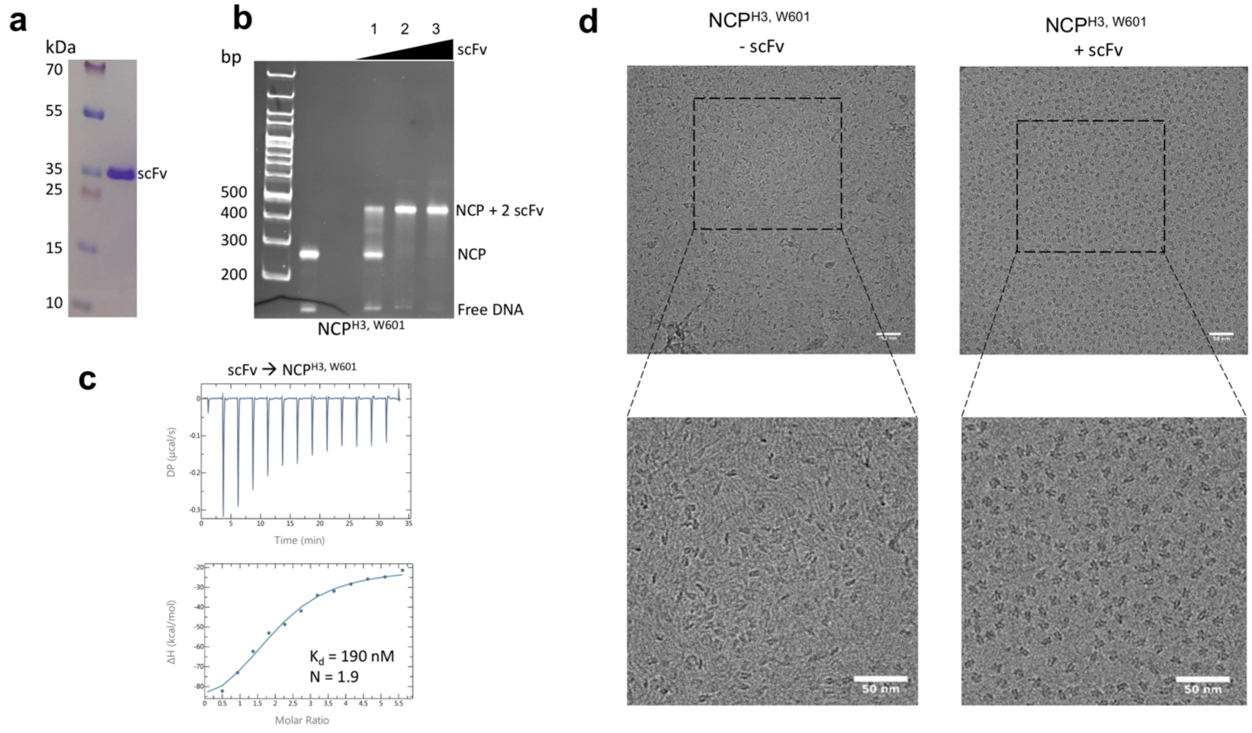

Supplementary Figure 1. Biochemical characterization of the interaction between scFv and NCP$^{H3, W601}$.

a. SDS PAGE gel of the scFv (29 kDa) after purification.

b. Electrophoretic mobility shift assay of scFv binding to NCP$^{H3, W601}$.

c. ITC profile of scFv titrated into NCP$^{H3, W601}$ solution, binding affinity and stoichiometry are indicated.

d. Representative cryo-EM images of NCP$^{H3, W601}$ without (left) and with scFv. Scale bar is 50 nm.

Uncropped gel images are provided as a Source Data file

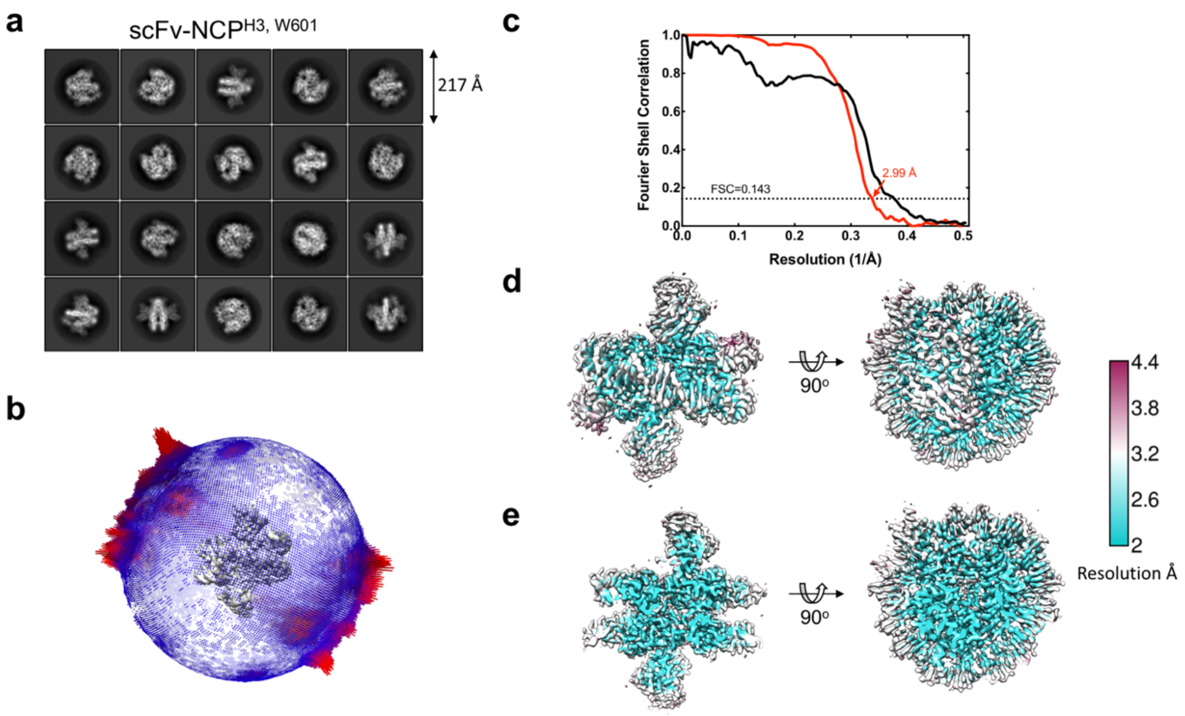

Supplementary Figure 2. Cryo-EM analysis of scFv-NCP$^{H3, W601}$

a. Representative 2D class averages of scFv-NCP$^{H3, W601}$ particles.

b. Euler angle distribution of particles used in the final 3D reconstruction, the height of the cylinder is proportional to the number of particles for that view.

c. Gold-standard Fourier shell correlation (FSC) curves of the final density map. Reported resolutions were based on the FSC=0.143 criteria. Red curve is scFv- CP $^{H3, W601}$ half map FSC and black curve is the model versus map FSC.

d. The final 3D density map of the scFv-NCP$^{H3, W601}$ complex shown in the indicated views is colored according to the local resolution estimated by the software ResMap.

e. The final half sliced 3D density map of the scFv-NCP$^{H3, W601}$ complex shown in the indicated views is colored according to the local resolution estimated by the software ResMap.

Data for the plot are provided as a Source Data file

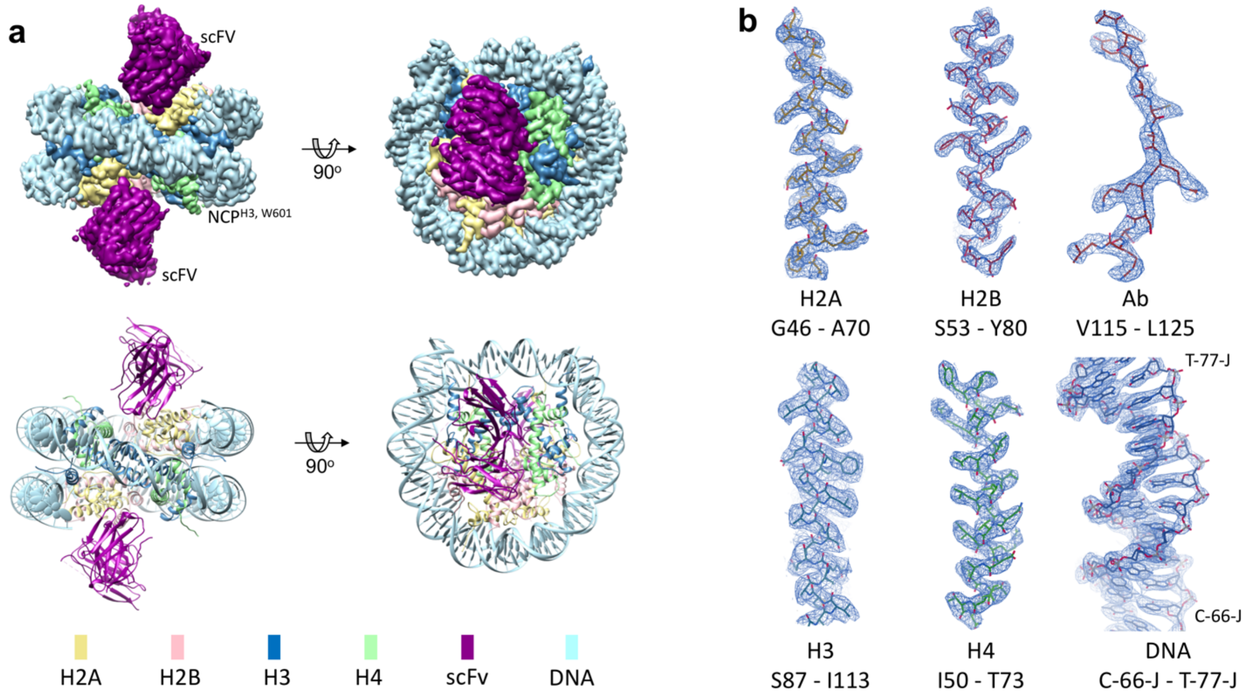

Supplementary Figure 3. Cryo-EM structure of the scFv-NCP$^{H3,\ W601}$ complex

a. Top, 3.0 Å resolution cryo-EM map of the scFv- NCP$^{H3,\ W601}$ complex. Left, side view; Right, top view. Color code: Light yellow, H2A; Pink, H2B; Blue, H3; Light green, H4; Dark magenta, scFv; light blue, DNA. Bottom: the fitted structure model.

b. Examples of cryo-EM densities map of histone proteins, scFv and DNA generated in Coot.

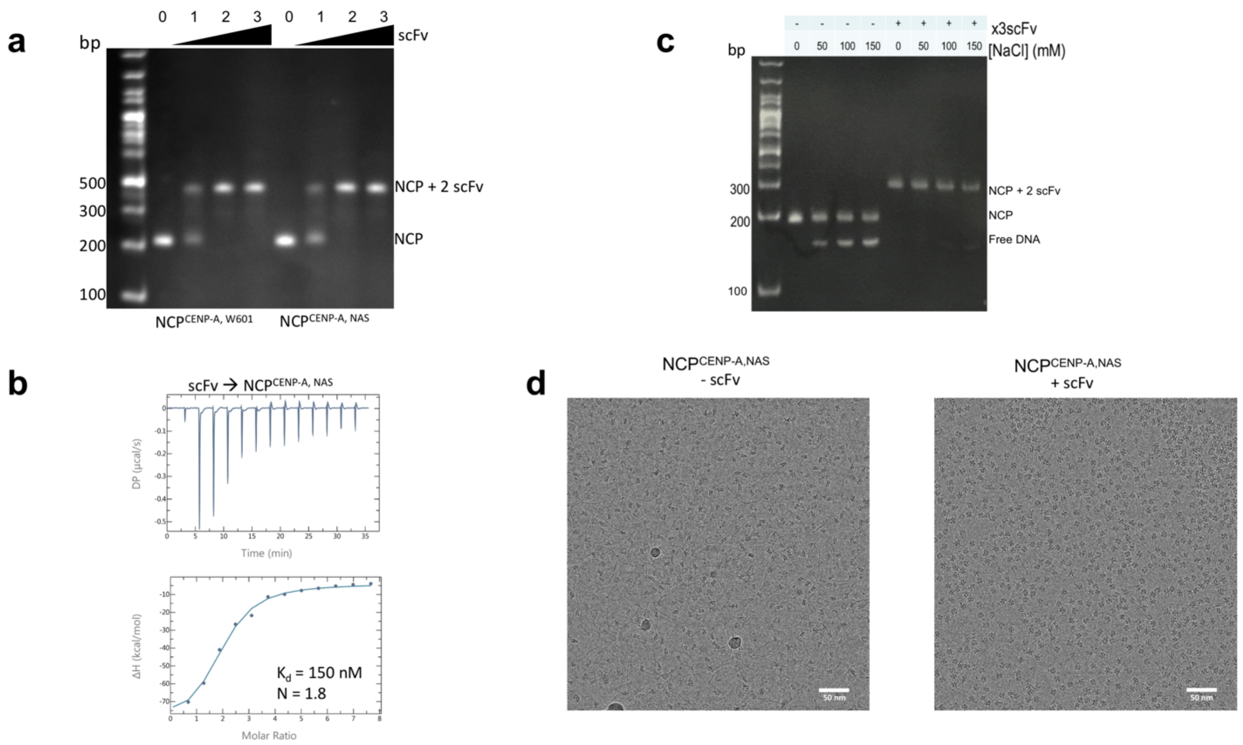

Supplementary Figure 4. Biochemical characterization of the interaction of scFv with the CENP-A nucleosome and stabilization analysis

a. Electrophoretic mobility shift assay of scFv binding to $NCP^{CENP\text{-}A,\ W601}$ (lane 2 – 5) or $NCP^{CENP\text{-}A,\ NAS}$ (lane 6 – 9).

b. ITC profile of scFv titrated into $NCP^{CENP\text{-}A,\ NAS}$ solution, binding affinities and stoichiometry are indicated.

c. Electrophoretic assay of $NCP^{CENP\text{-}A,\ NAS}$ (lane 2 – 5) or scFv-$NCP^{CENP\text{-}A,\ NAS}$ complex (lane 6 – 9) at various salt concentration.

d. Representative cryo-EM images of $NCP^{CENP\text{-}A,\ NAS}$ without (left) and with (right) scFv. Scale bar is 50 nm.

Uncropped gel images are provided as a Source Data file

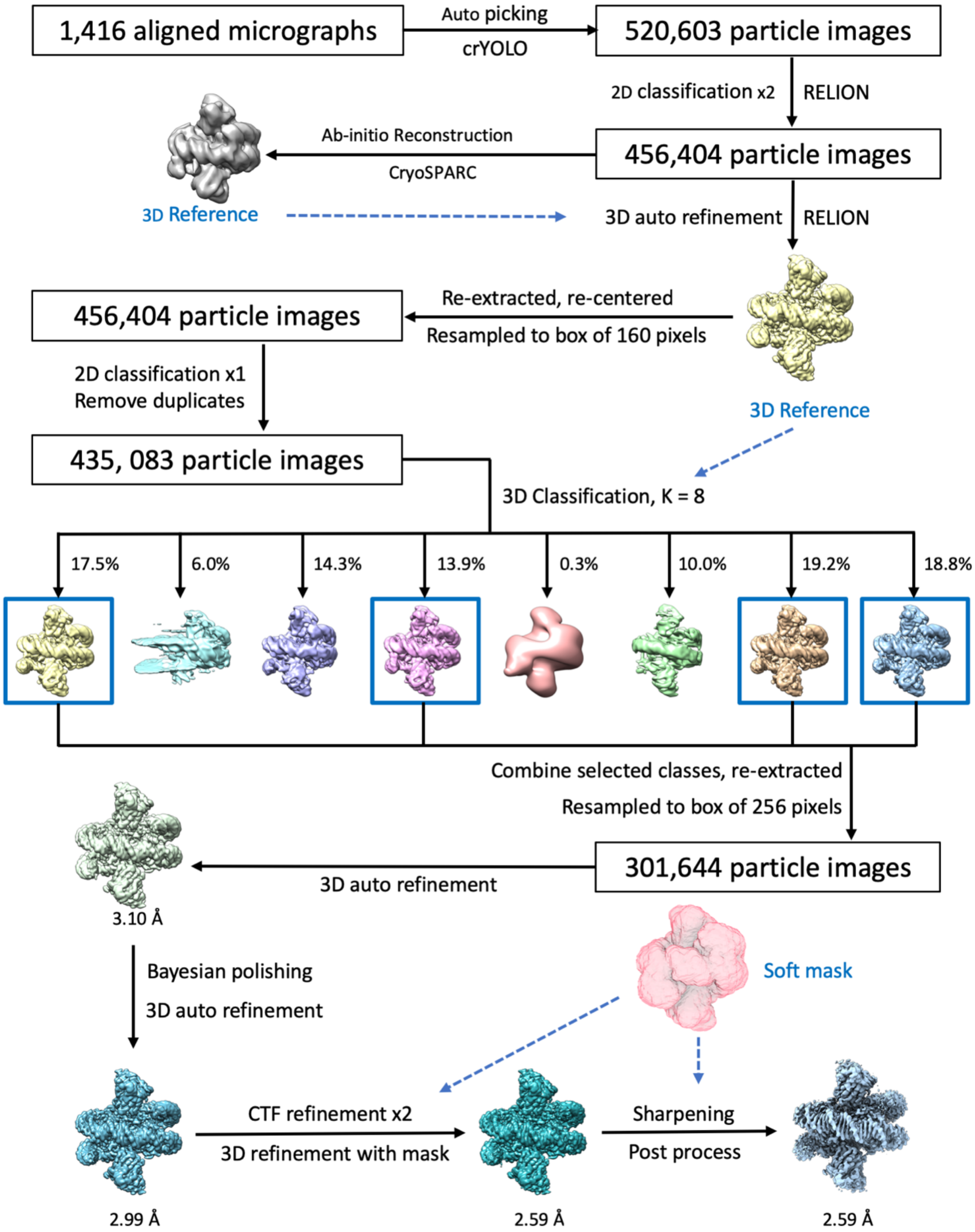

Supplementary Figure 5. Flowchart of data processing of scFv-NCP$^{CENP\text{-}A,\ NAS}$

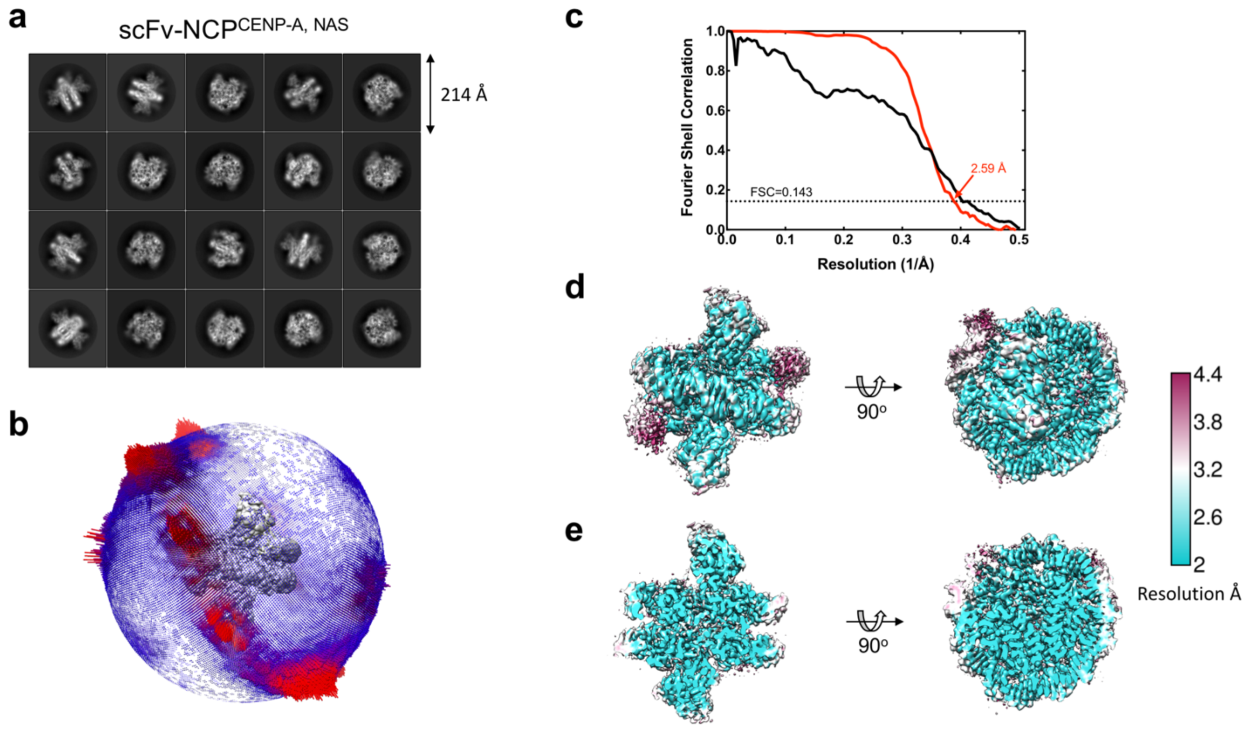

Supplementary Figure 6. Cryo-EM analysis of scFv-NCP$^{CENP\text{-}A,\ NAS}$

a. Representative 2D class averages of scFv-NCP$^{CENP\text{-}A,\ NAS}$ particles.

b. Euler angle distribution of particles used in the final 3D reconstruction, the height of the cylinder is proportional to the number of particles for that view.

c. Gold-standard Fourier shell correlation (FSC) curves of the final density map. Reported resolutions were based on the FSC=0.143 criteria. Red curve is the scFv-NCP $^{CENP\text{-}A,\ NAS}$ FSC of two independent half maps and black curve is the structural model versus summed map FSC calculated by RELION.

d. The final 3D density map of scFv-NCP$^{CENP\text{-}A,\ NAS}$ shown in the indicated views is colored according to the local resolution estimated by the software ResMap.

e. The final half sliced 3D density map of scFv-NCP$^{CENP\text{-}A,\ NAS}$ shown in the indicated views is colored according to the local resolution estimated by the software ResMap.

Data for the plot are provided as a Source Data file

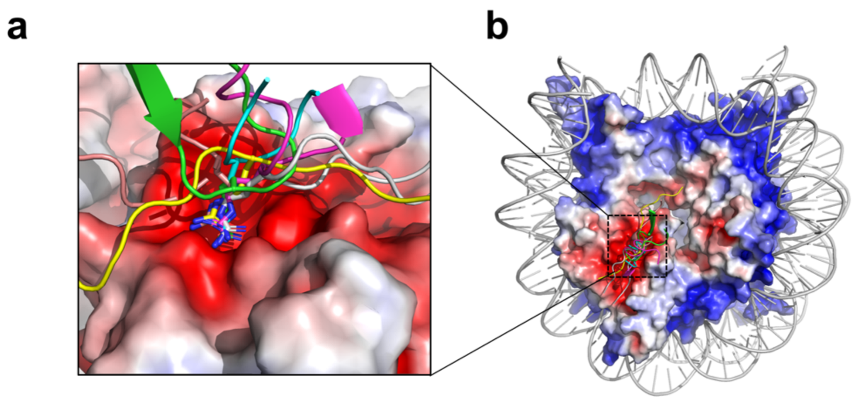

c

scFv	CARKSSRLRST-LDYW
CENP-C motif	RRSNRIRLKPLDYW
CENP-C central	SRRISRRPSDWWVV
LANA	MAPPGMRLRSGRSTG
HMGNs	RRSARLSA
Sir3	NNRRRSRKRGGE
RCC1	GEGRRGK
PRC1	KKLVSKRSLR

Supplementary Figure 7. Various nucleosome binding proteins share the same "Arginine" anchor that inserts into the acidic patch pocket.

a. Close-up view of various nucleosome binding proteins interacting with acidic patch. The anchoring arginine side chains from scFv (green), CENP-C motif (yellow), LANA (grey), Sir3(magenta), RCC1(cyan), and PRC1(pink) are illustrated in sticks, and the proteins are shown as cartoons.

b. The regions of scFv, CENP-C motif, LANA, Sir3, RCC1, and PRC1 that interact with nucleosome acidic patch are overlaid together. Nucleosome histone octamer is shown with surface electrostatic potential view. DNA is shown as cartoon.

c. Local sequences of proteins that bind to the acidic patch. Alignment was made using the anchoring arginine residues of scFv, CENP-C motif and central region, LANA, HMGN, Sir3, RCC1, and PRC1.

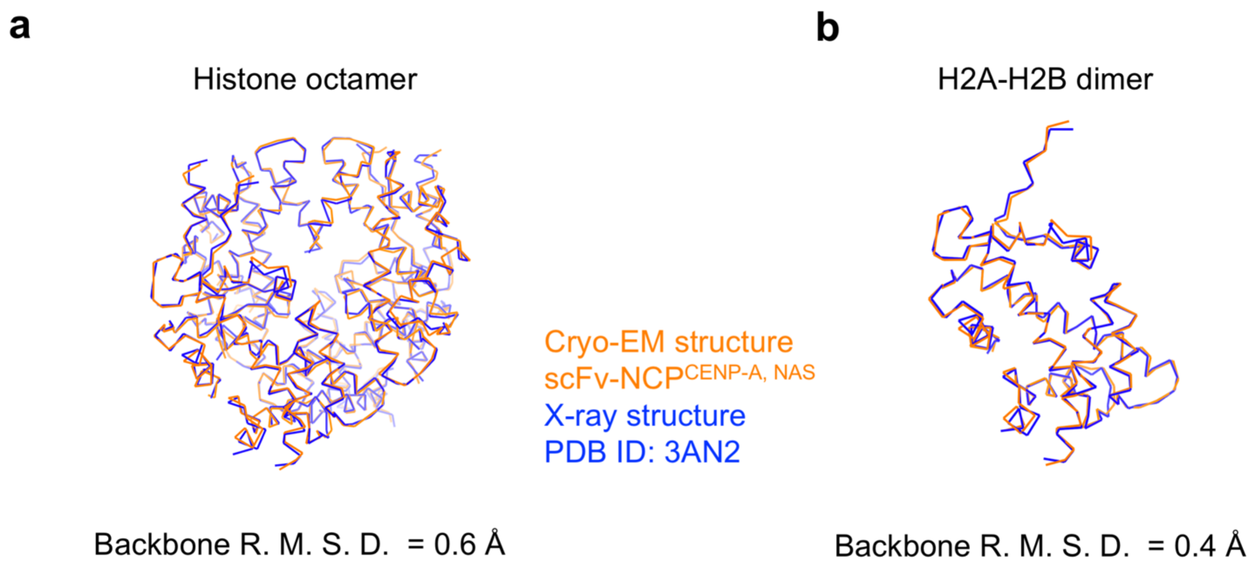

Supplementary Figure 8. Structure of the core histone proteins is not affected by scFv binding.

a. Alignment of the structures of all core histones in NCP$^{CENP\text{-}A,NAS}$ and the NCP$^{CENP\text{-}A,PAS}$ crystal structure (PDB ID: 3AN2).

b. Alignment of the structures of core histones H2A and H2B in NCP$^{CENP\text{-}A,NAS}$ and the NCP$^{CENP\text{-}A,PAS}$ crystal structure (PDB ID: 3AN2).

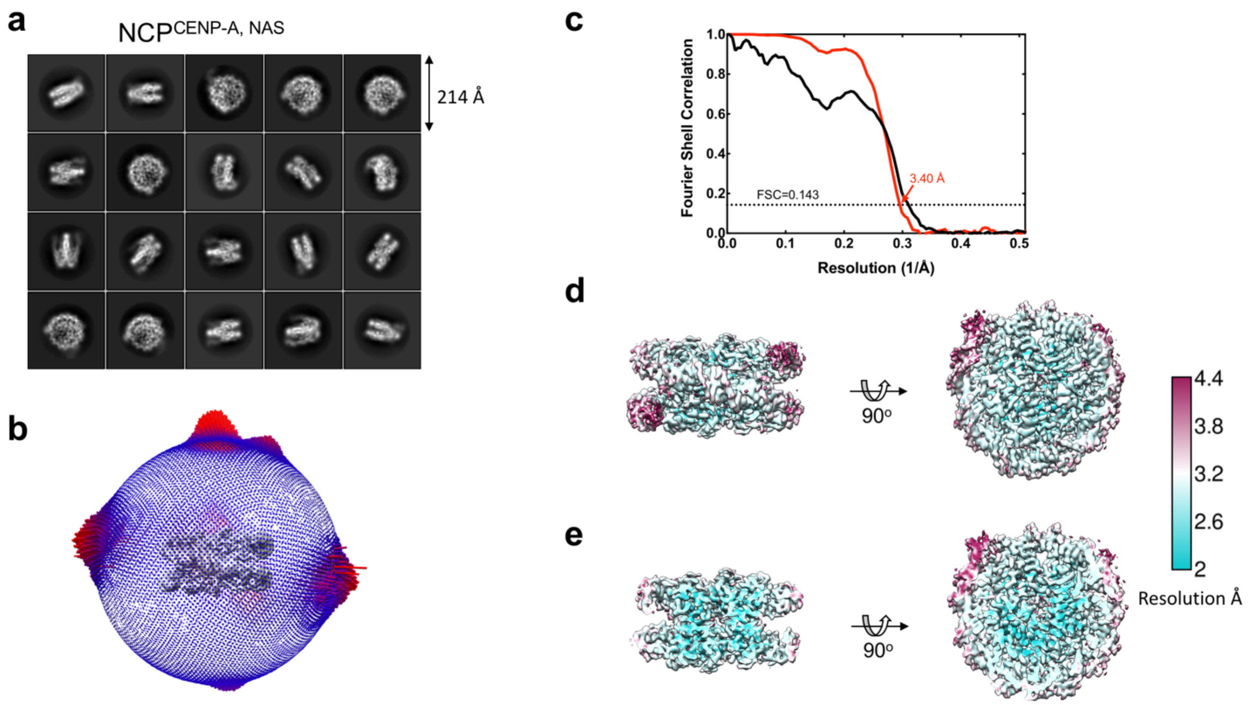

Supplementary Figure 9. Cryo-EM analysis of NCP$^{\text{CENP-A, NAS}}$

a. Representative 2D class averages of NCP$^{\text{CENP-A, NAS}}$ particles.

b. Euler angle distribution of particles used in the final 3D reconstruction, the height of the cylinder is proportional to the number of particles for that view.

c. Gold-standard Fourier shell correlation (FSC) curves of the final density map. Reported resolutions were based on the FSC=0.143 criteria. Red curve is the scFv-NCP $^{\text{CENP-A, NAS}}$ FSC of two independent half maps and black curve is the structural model versus summed map FSC calculated by RELION.

d. The final 3D density map of NCP$^{\text{CENP-A, NAS}}$ shown in the indicated views is colored according to the local resolution estimated by the software ResMap.

e. The final half sliced 3D density map of scFv-NCP$^{\text{CENP-A, NAS}}$ shown in the indicated views is colored according to the local resolution estimated by the software ResMap.

Data for the plot are provided as a Source Data file.

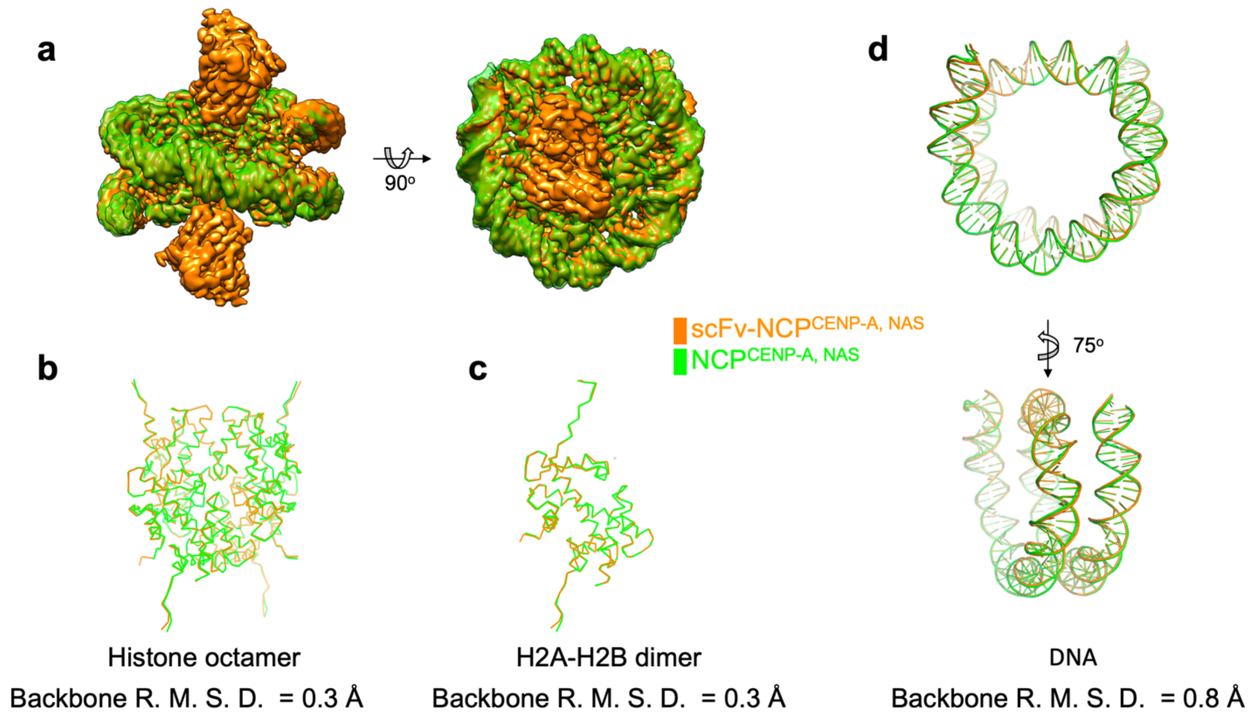

Supplementary Figure 10. scFv does not perturb the structure of NCP$^{CENP-A, NAS}$

a. Overlay the unsharpened cryo-EM maps of scFv-NCP$^{CENP-A, NAS}$ and the free NCP$^{CENP-A, NAS}$

b. Alignment of the structures of all core histones in scFv-NCP$^{CENP-A,NAS}$ and the free NCP$^{CENP-A,NAS}$

c. Alignment of the structures of core histones H2A and H2B in scFv-NCP$^{CENP-A,NAS}$ and the free NCP$^{CENP-A,NAS}$

d. Alignment of the structures of DNA in scFv-NCP$^{CENP-A,NAS}$ and the free NCP$^{CENP-A,NAS}$

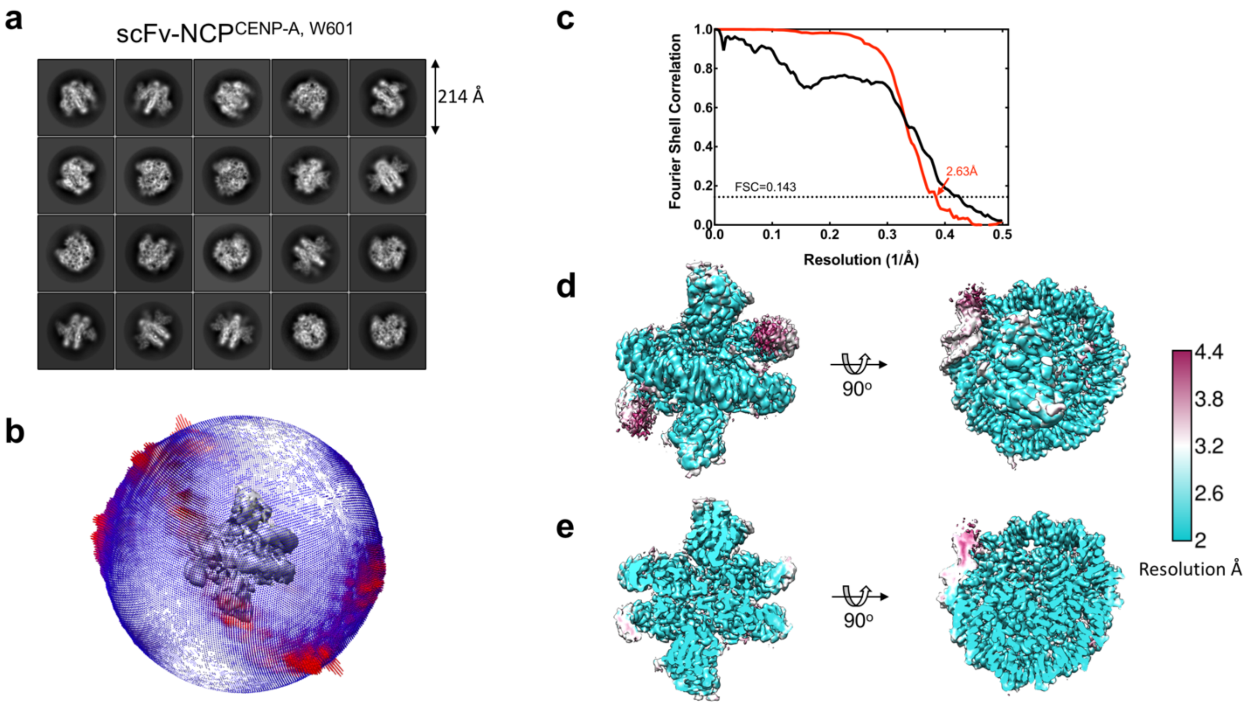

Supplementary Figure 11. Cryo-EM analysis of the scFv-NCP$^{CENP\text{-}A,\ W601}$ complex.

a. Representative 2D class averages of scFv-NCP$^{CENP\text{-}A,\ W601}$ particles.

b. Euler angle distribution of particles used in the final 3D reconstruction, the height of the cylinder is proportional to the number of particles for that view.

c. Gold-standard Fourier shell correlation (FSC) curves of the final density map. Reported resolutions were based on the FSC=0.143 criteria. Red curve is the scFv-NCP$^{CENP\text{-}A,\ W601}$ half map FSC and black curve is the model versus map FSC.

d. The final 3D density map of scFv-NCP$^{CENP\text{-}A,\ W601}$ shown in the indicated views is colored according to the local resolution estimated by the software ResMap.

e. The final half sliced 3D density map of scFv-NCP$^{CENP\text{-}A,\ W601}$ shown in the indicated views is colored according to the local resolution estimated by the software ResMap.

Data for the plot are provided as a Source Data file.

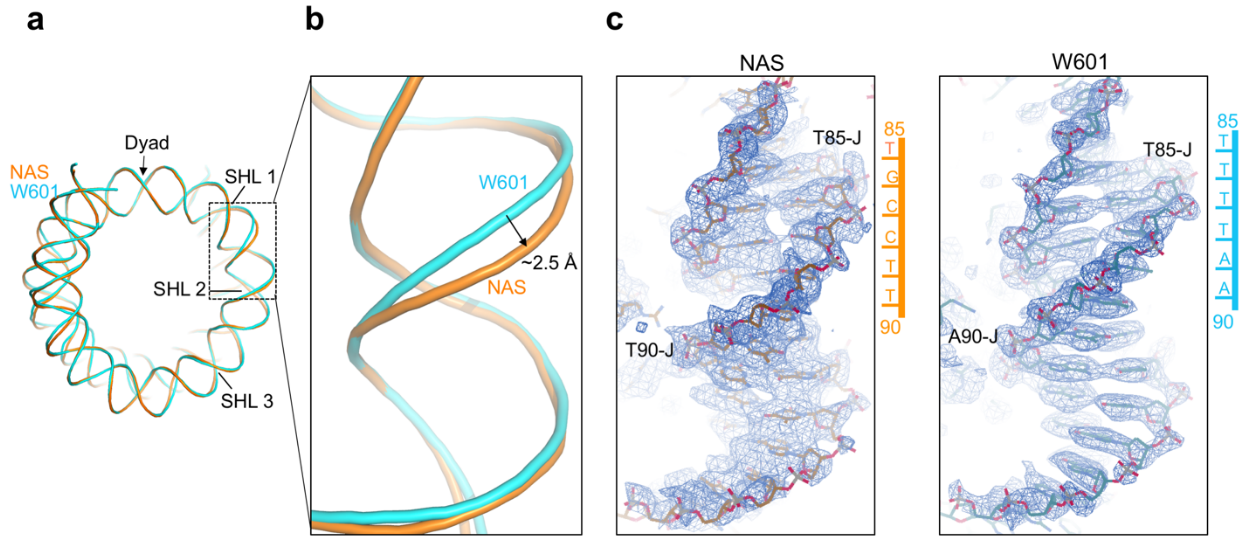

Supplementary Figure 12. Comparison of the DNA conformation in scFv-NCP$^{CENP\text{-}A,\ NAS}$ and scFv-NCP$^{CENP\text{-}A,\ W601}$.

a. Alignment of the DNA in scFv-NCP$^{CENP\text{-}A,\ NAS}$ and scFv-NCP$^{CENP\text{-}A,\ W601}$.

b. Enlarged region from SHL 1 to SHL 2.

c. Density maps corresponding to regions from SHL 1 to SHL 2.

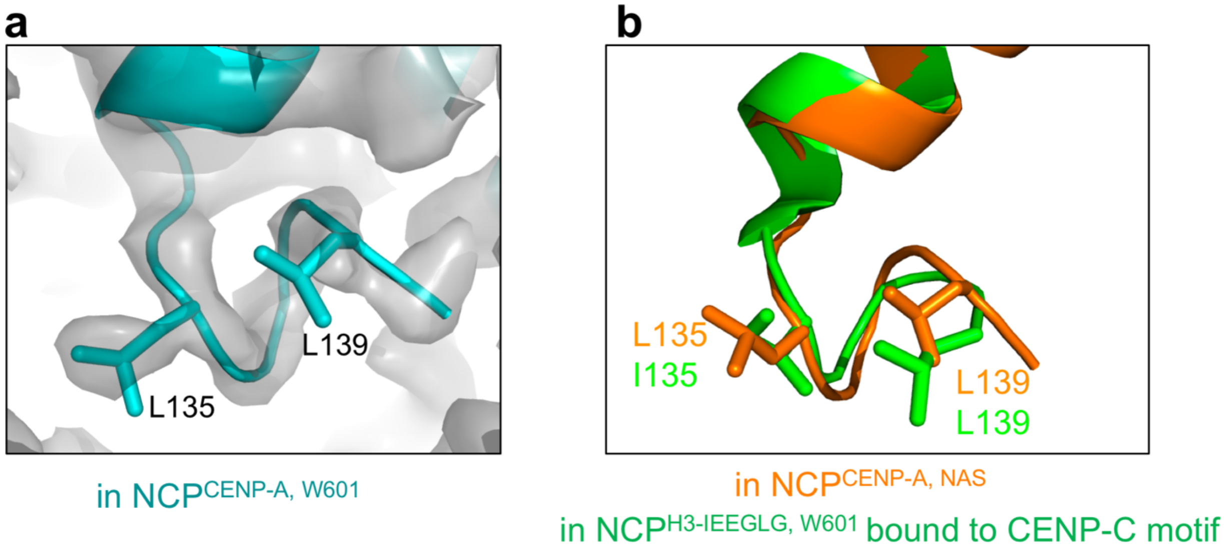

Supplementary Figure 13. Conformation of the C-terminal tail of CENP-A.

a. Density map in the CENP-A C-terminal tail region in scFv-NCP$^{\text{CENP-A, W601}}$.

b. Structural alignment of CENP-A in scFv-NCP$^{\text{CENP-A, NAS}}$ and H3-IEEGLG in the NCP$^{\text{H3-IEEGLG, W601}}$ bound to CENP-C motif (PDB ID: 4X23).

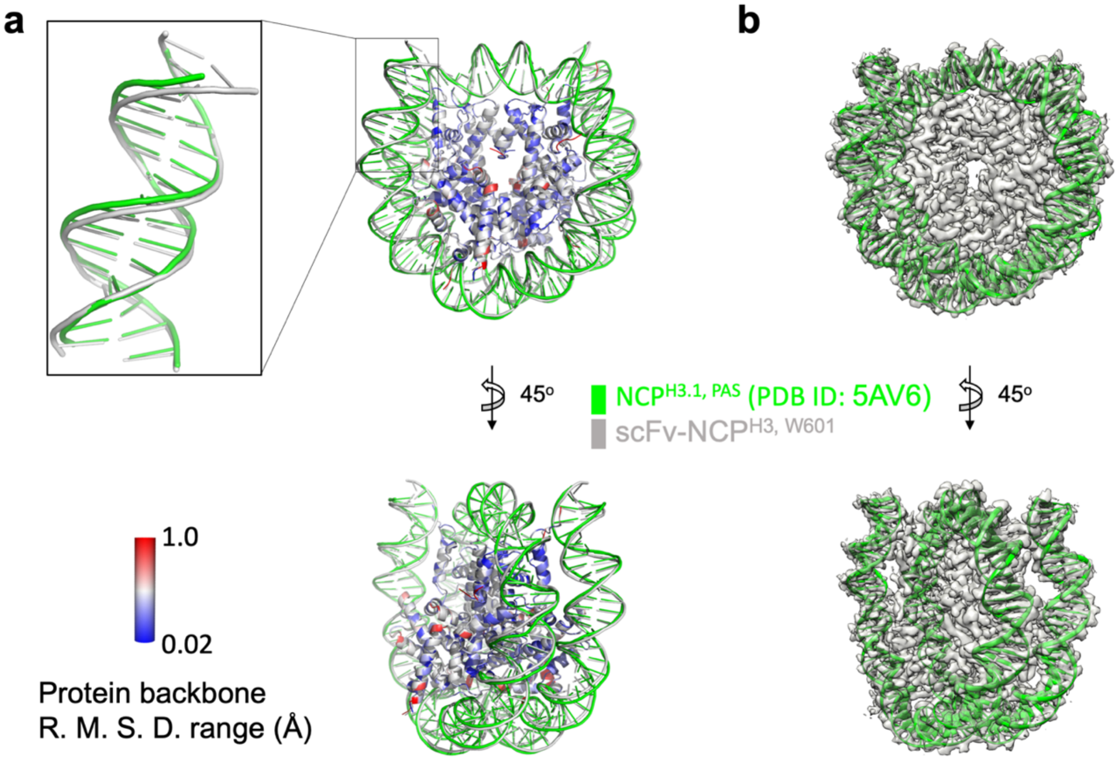

Supplementary Figure 14. Comparison between crystal structure and cryo-EM structure of NCP with histone H3.

a. Alignment of the crystal structure of $NCP^{H3.1, PAS}$ (PDB ID: 5AV6) and the Cryo-EM structure $scFv\text{-}NCP^{H3, W601}$. Histone octamer of $NCP^{H3.1, PAS}$ is colored according to the local R. M. S. D. between two structures. The blue-white-red spectrum bar shows the range of local R. M. S. D. value of protein backbone atoms from 0.02 to 1.0 Å. DNA in $NCP^{H3, PAS}$ structure is colored in green and $scFv\text{-}NCP^{H3, W601}$ structure is colored in light grey. Enlarged panel shows that the backbones of the DNA ends in the two structures display similar closed conformation.

b. $NCP^{H3.1, PAS}$ structure (PDB ID: 5AV6) can be fitted well into the cryo-EM density map of $scFv\text{-}NCP^{H3, W601}$. Only DNA ribbon structure (green) is shown. scFv densities are masked out for clarity. Figure is prepared in Chimera with map level at 0.0146.

Supplementary Table 1 | Cryo-EM data collection, refinement and validation statistics

	scFv-NCP^{H3,W601} (PDB 6DZT) (EMD-8938)	scFv-NCP$^{CENP\text{-}A,W601}$ (PDB 6E0C) (EMD- 8945)	scFv-NCP$^{CENP\text{-}A, NAS}$ (PDB 6E0P) (EMD- 8949)	NCP$^{CENP\text{-}A, NAS}$ (PDB 6O1D) (EMD- 0586)
**Data collection and processing**				
Magnification	14k	18k	18k	18k
Voltage (kV)	300	300	300	300
Electron exposure (e-/Å^2)	40	40	40	40
Defocus range (μm)	0.8-2.0	0.8-1.6	0.8-1.6	0.8-1.6
Physical pixel size (Å)	1.72	1.358	1.358	1.358
Symmetry imposed	C1	C1	C1	C1
Initial particle images (no.)	765,253	629,392	520,603	841,392
Final particle images (no.)	238,790	494,510	301,644	303,863
Map resolution (Å)	2.99	2.63	2.59	3.40
FSC threshold	0.143	0.143	0.143	0.143
Map resolution range (Å)	2.0 – 6.9	2.0 – 4.0	2.0 – 4.0	2.6 – 6.8
**Refinement**				
Initial model used (PDB code)	3LZ0, 1KX5, 2GKI	3AN2	3AN2	6E0P
Model vs map resolution (Å)	3.10	2.92	3.12	3.68
FSC threshold	0.5	0.5	0.5	0.5
Map sharpening *B* factor (Å^2)	-63	-20	-24	-80
Model composition				
Nucleic acid (bp)	147	147	145	145
Protein residues	1213	1220	1220	671
R.m.s. deviations				
Bond lengths (Å^2)	0.01	0.008	0.007	0.01
Bond angles (°)	0.885	0.783	0.863	0.922
Validation				
MolProbity score	1.23	1.39	1.33	1.18
Clashscore	2.08	2.04	2.41	1.96
Poor rotamers (%)	0.00	0.19	0.00	0.00
Ramachandran plot				
Favored (%)	96.30	95.74	95.57	96.65
Allowed (%)	3.70	4.26	4.43	3.35
Disallowed (%)	0.00	0.00	0.00	0.00
