# Supplementary material for: Atomic resolution cryo-EM structure of a native-like CENP-A nucleosome aided by an antibody fragment
Source: Nat Commun. 2019 May 24;10:2301. doi: 10.1038/s41467-019-10247-4 (PMC6534667; doi:10.1038/s41467-019-10247-4)
Supplement: Supplementary file 3 — Description of Additional Supplementary Files [file 41467_2019_10247_MOESM3_ESM.pdf]

Description of Additional Supplementary Files

File name: Supplementary Movie 1

Description: Cryo-EM map and structural features of human CENP-A nucleosome bound to the scFv.
